# Twenty-two novel glacier *Pseudomonas* species expand cultivated representation: nitrogen-cycling potential and temperature-responsive proteomes

**DOI:** 10.1093/ismeco/ycag204

**Published:** 2026-07-12

**Authors:** Chan Zhao, Lei-Lei Yang, Yu-Hua Xin, Qing Liu

**Affiliations:** State Key Laboratory of Microbial Diversity and Innovative Utilization, Institute of Microbiology, Chinese Academy of Sciences, Beijing 100101, China; State Key Laboratory of Animal Biotech Breeding and College of Biological Sciences, China Agricultural University, Beijing 100193, P. R. China; China General Microbiological Culture Collection Center (CGMCC), Institute of Microbiology, Chinese Academy of Sciences, Beijing 100101, P. R. China; State Key Laboratory of Microbial Diversity and Innovative Utilization, Institute of Microbiology, Chinese Academy of Sciences, Beijing 100101, China; China General Microbiological Culture Collection Center (CGMCC), Institute of Microbiology, Chinese Academy of Sciences, Beijing 100101, P. R. China; State Key Laboratory of Microbial Diversity and Innovative Utilization, Institute of Microbiology, Chinese Academy of Sciences, Beijing 100101, China; China General Microbiological Culture Collection Center (CGMCC), Institute of Microbiology, Chinese Academy of Sciences, Beijing 100101, P. R. China; State Key Laboratory of Microbial Diversity and Innovative Utilization, Institute of Microbiology, Chinese Academy of Sciences, Beijing 100101, China; China General Microbiological Culture Collection Center (CGMCC), Institute of Microbiology, Chinese Academy of Sciences, Beijing 100101, P. R. China; Beijing Key Laboratory of Genetic Element Biosourcing & Intelligent Design for Biomanufacturing, Institute of Microbiology, Chinese Academy of Sciences, Beijing 100101, P. R. China

**Keywords:** cryosphere microbiology, glacier bacteria, *Pseudomonas,* genome-based taxonomy, nitrogen-transformation potential, temperature-responsive proteomics, cultivated diversity

## Abstract

Accelerating glacier retreat exports cryospheric microbiomes into downstream ecosystems, raising questions about their functional roles. However, a persistent gap between genomic predictions and physiologically validated functions exists, largely due to the scarcity of cultivated and taxonomically resolved model organisms from these environments. Here we characterize 23 *Pseudomonas* strains isolated from ice, cryoconite, and supraglacial meltwater collected across ten glaciers in China. Phylogenomic inference places them within the *Pseudomonas mandelii* subgroup, and genome metrics delineate 22 strains as distinct novel species (ANI ≤95.71%; dDDH ≤64.6% to their closest type strains). Phenotypic analyses showed broad environmental tolerance, including growth at −2°C, strain-dependent upper growth limits of 30°C–37°C, and growth over an approximate pH range of 5.0–11.0. Genome-based reconstruction predicted heterogeneous nitrogen-transforming potential, including DNRA-related genes in all strains, nitrate-reduction genes in 14 strains, complete denitrification marker sets in 12 strains, and nitrogen-fixation genes in one strain. Phenotypic assays showed broader nitrate-reduction activity than predicted, with all 23 strains reducing nitrate to nitrite and seven showing reduction beyond nitrite, underscoring the value of cultivation-based functional validation in non-model cryospheric bacteria. Comparative proteomics of four representative species at 0°C versus 28°C reveals shared temperature-responsive pathways and a common network backbone involving translation, proteostasis, and carbon economy. Together, these cultured genomes provide valuable models for linking cryospheric *Pseudomonas* diversity to temperature-responsive physiology and biogeochemical potential.

## Introduction

Glaciers are a major component of the terrestrial cryosphere and sustain diverse, metabolically active microbial communities despite chronic cold, intense UV exposure, oligotrophy, and repeated freeze–thaw cycles. Cryospheric microbiomes encompass bacteria, archaea, fungi, algae, and viruses, and bacterial assemblages are frequently dominated by *Pseudomonadota*, *Actinobacteria*, and *Bacteroidota* [1–6]. Persistence in these habitats relies on compatible-solute accumulation, increased membrane unsaturation, cold-shock and antifreeze proteins, and biofilm formation that can enhance nutrient retention and buffer environmental fluctuations [7, 8]. China contains one of the largest glacierized regions outside the polar areas, with glaciers concentrated mainly in western China rather than being evenly distributed across the country. These glaciers occur across major high-mountain systems, including the Tibetan Plateau and surrounding ranges such as the Tien Shan, Qilian Mountains, Pamir–Karakoram–Himalayan region, and Hengduan Mountains [5]. They represent important freshwater reserves and climate-sensitive habitats, and their retreat is expected to reshape microbial dispersal, nutrient cycling, and downstream ecosystem succession.

Within cryospheric communities, *Pseudomonas* is a widely distributed bacterial group noted for broad metabolic potential and physiological plasticity [9]. As of February 2026, the genus *Pseudomonas* comprises 362 species with validly published names, reflecting extensive diversification across habitats [10]. Several cold-environment *Pseudomonas* strains express traits consistent with cryoprotection and low-temperature activity, including polyhydroxyalkanoate accumulation and cold-active enzymes [11, 12]. Although *Pseudomonas* is frequently detected in glacier amplicon and metagenomic surveys, cultivated cryospheric *Pseudomonas* remain limited, constraining genotype–phenotype linkage and tests of strain-level contributions to ecosystem function. For example, *Pseudomonas* reached relative abundances of up to 1.1% in the Chongce Ice Cap [13], and supraglacial metagenomes from Matanuska Glacier contained multiple *Pseudomonas* among highly represented operational taxonomic units [14]. These observations motivate the cultivation of phenotypically well-resolved isolates to link phylogeny and genomes to traits that sustain activity under extreme conditions. Beyond genome-based inference, systems-level measurements such as comparative proteomics can identify temperature-responsive pathways and functional modules that are not readily predictable from sequence data alone [7, 15].

Glacier retreat is accelerating globally, increasing connectivity among previously isolated habitats and exporting cryospheric cells and extracellular DNA into forefields and downstream waters, with consequences for early successional assembly and biogeochemical processing [6]. Reports of antibiotic resistance- and virulence-associated genetic markers in cryospheric and forefield microbiomes raise questions about the extent to which such signatures may be mobilized with meltwater [16]. Glacier microbiomes also contribute to elemental cycling, including nitrogen transformations, with community structure and functional potential varying across climatic regimes [17].

In the present study, cultivation was not designed to selectively target *Pseudomonas*. Rather, the strains analyzed here were selected from a broader collection of bacteria isolated from glacier ice, cryoconite, and supraglacial meltwater across ten glaciers in western China. Preliminary 16S rRNA gene identification showed that *Pseudomonas* was the most abundant genus in this cultured collection, accounting for 11.4% of all isolates, which motivated a focused genus-level analysis. Using phylogenomics and polyphasic taxonomy, we demonstrate that these isolates comprise 22 novel species within the *Pseudomonas mandelii* subgroup. We further apply genome-enabled functional profiling, nitrate-reduction assays, and comparative proteomics to evaluate their nitrogen-transformation potential, genome–phenotype consistency, and temperature-responsive physiology. These data expand cultivated cryospheric *Pseudomonas* diversity and provide experimentally tractable models for investigating microbial function in rapidly changing glacier ecosystems.

## Materials and methods

Samples (cryoconite, ice, supraglacial meltwater) were collected from ten glaciers in western China (Table S1;  Fig.  S1). To recover a broad range of cultivable glacier-derived bacteria, samples were plated on both PYG agar [18] and R2A agar. PYG agar was used as a relatively nutrient-rich medium to support heterotrophic colony formation, whereas R2A agar was included as a lower-nutrient medium commonly used for environmental bacteria. Plates were incubated at 14°C to facilitate visible colony formation within a practical time frame while maintaining a low-temperature cultivation regime suitable for recovering cold-adapted bacteria. Distinct colonies were purified by repeated streaking. The 16S rRNA genes were amplified using primers 27F/1492R [19]. The purified PCR products were bidirectionally sequenced using an ABI 3730xl DNA Analyzer (Applied Biosystems, Foster City, CA, USA) at Beijing Sinogenomax Co., Ltd. The resulting sequences were assembled and compared with the EzBioCloud database [20]. Sequences were aligned with MAFFT v7.490 [21] and analyzed by Neighbor-Joining in MEGA v12 with 1000 bootstrap replicates [22]. In total, approximately 3996 bacterial isolates were obtained from the glacier samples. Preliminary taxonomic identification based on 16S rRNA gene sequencing assigned 458 isolates to the genus *Pseudomonas*, representing 11.4% of the cultured collection and making *Pseudomonas* the most abundant genus recovered. Twenty-three strains representing distinct phylogenetic lineages and putative novel species were selected for whole-genome sequencing, genome-wide relatedness analysis, and polyphasic taxonomic characterization.

Genomes were generated using PacBio Sequel II long reads and Illumina paired-end short reads. Unicycler v0.5.1 was used as the primary hybrid assembler because it integrates PacBio long reads and Illumina short reads [23]. When Unicycler did not generate a closed or sufficiently continuous assembly, Flye v2.95 [24] long-read assemblies polished with Pilon v1.24 [25] were used instead. Assembly quality was assessed with CheckM2 v1.0.2 [26] and QUAST v5.3.0 [27]. For each strain, the assembly retained for downstream analyses was selected based on overall assembly quality, including contiguity, completeness, contamination, and genome structure. General genome annotation was performed using Bakta v1.10.3 with its associated reference database [28]. Phylogenomics used UBCG2 v2.0 [29], MAFFT v7.490 alignments, Gblocks v0.91b trimming [30], and IQ-TREE v2.0.7 [31]. Average nucleotide identity (ANI) and digital DNA–DNA hybridization (dDDH) were computed with FastANI v1.34 [32] and TYGS [33]. Element cycling potentials were assessed with METABOLIC v4.0 [34] based on its marker-gene annotation workflow. Antibiotic resistance genes (ARGs) and virulence factor genes (VFGs) were screened with ABRicate v1.2.0 [35] against CARD, ResFinder, NCBI AMR, ARG-ANNOT, MEGARes, VFDB, and Victors.

Phenotypic characterization followed the procedures described previously [18], with key conditions summarized here. Growth temperature was tested at −2, 0, 4, 10, 14, 20, 25, 28, 30, 32, 37, and 40°C. Incubation at −2°C was performed in a temperature-controlled low-temperature incubator. pH tolerance was examined over pH 4.0–11.0 at 1.0-unit intervals, and NaCl tolerance was tested from 0% to 5.0% (w/v). Carbon source utilization was tested using API 50 CH strips (bioMérieux) with a basal medium [18]. Other phenotypic tests including nitrate reduction were assessed using API 20NE, API 20E, and API ZYM strips (bioMérieux) according to the manufacturer’s instructions.

For proteomics, four strains were selected to represent major phylogenetic branches within the dataset. Proteomic comparisons were performed at 0°C and 28°C; 0°C was used as a standardized near-freezing condition that allowed stable shaking cultivation and reproducible sampling of exponential-phase cells across strains. Cells were harvested in the exponential phase, analyzed by LC–MS/MS using dia-PASEF, and processed with DIA-NN v1.8 [36, 37]. Differential abundance was tested using limma [38]. Proteins were considered temperature responsive at a Benjamini–Hochberg FDR-adjusted *P* < .05 and an absolute fold change ≥3. KEGG-based functional summaries and enrichment were assessed using Fisher’s exact tests. Protein association networks were constructed using STRING (v12.0). Networks from the four strains were unified by gene name, and edges were retained when supported by at least three strains [39].

## Results

### Genome-resolved placement and species-level novelty

All 23 isolates were assigned to the genus *Pseudomonas* based on 16S rRNA gene sequences. Pairwise 16S rRNA gene similarities among them ranged from 96.6–100%, and each isolate shared ≥99.1% similarity with at least one described *Pseudomonas* species (Table S2). These high similarities reflect the limited resolution of the 16S rRNA gene within *Pseudomonas* [40, 41], thus necessitating genome-based species delimitation.

**Figure 1:**
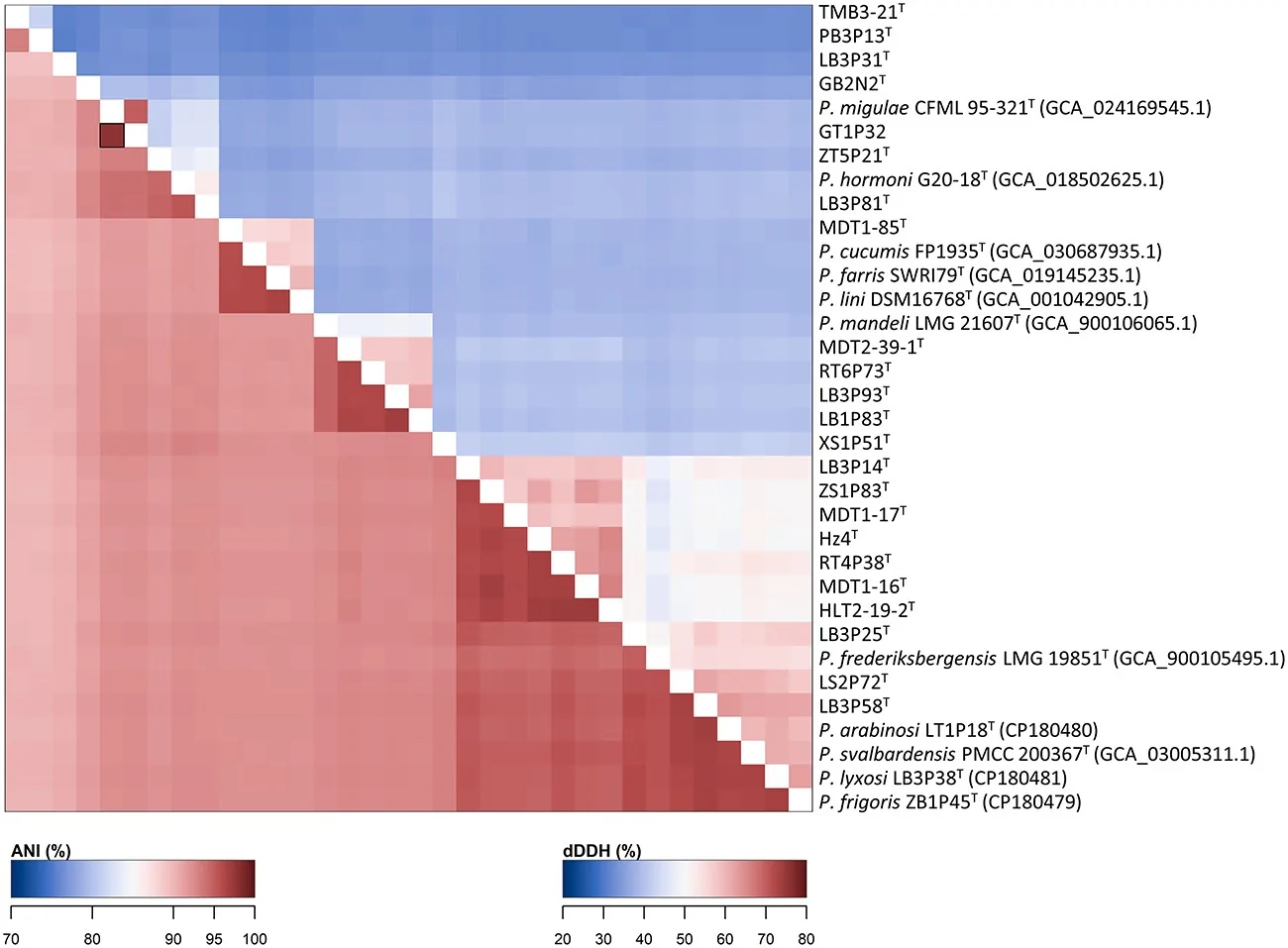
Genome-wide relatedness of glacier-derived *Pseudomonas* isolates to closely related type strains. Dual-panel heatmap illustrating pairwise genomic relatedness. The lower-left triangle displays ANI values, while the upper-right triangle displays dDDH values between the 23 isolates and 11 reference type-strain genomes. Two independent color scales represent ANI and dDDH values. The predominantly sub-threshold values across both metrics provide congruent genomic evidence that 22 isolates represent species-level lineages distinct from currently named species. Strain GT1P32 shows the highest genome-wide relatedness to *P. migulae* CFML 95-321^T^ and lies near the commonly used species boundary, consistent with its interpretation as a borderline strain affiliated with *P. migulae* rather than as a clearly distinct novel species.

**Figure 2:**
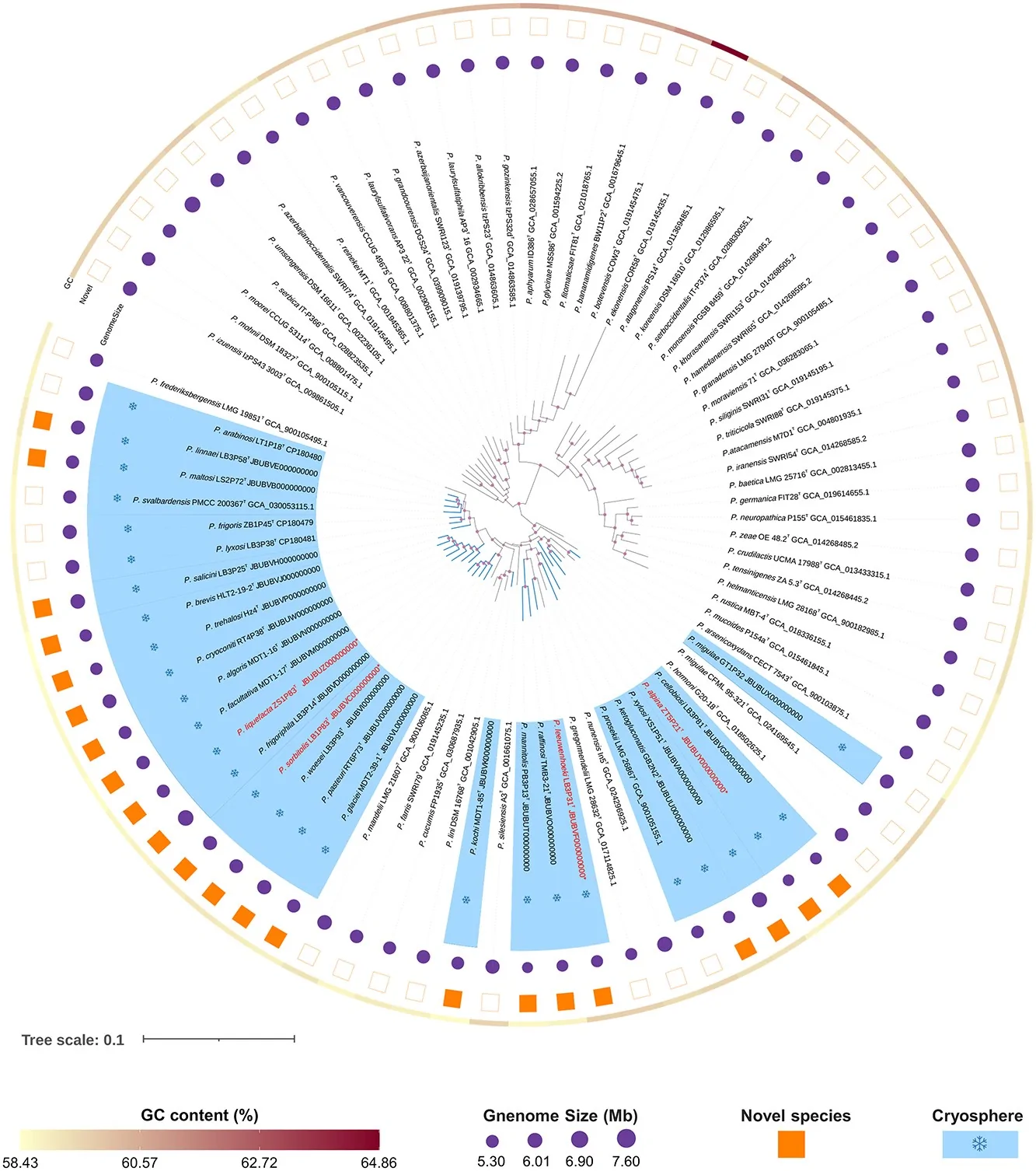
Phylogenomic placement of the 23 glacier-derived *Pseudomonas* isolates within the *P. mandelii* subgroup. Maximum-likelihood phylogeny inferred from a concatenated core-gene alignment including the 23 isolates and closely related taxa. The tree shown here represents a subtree extracted from a broader genus-level *Pseudomonas* phylogenomic analysis; taxa were selected to include the *P. mandelii* subgroup and the closest type-strain relatives of the glacier-derived isolates for clearer visualization of their taxonomic placement. GTR + F + I + R9 was applied as the best-fit nucleotide substitution model. The 22 proposed novel species form well-supported, independent lineages relative to currently named species, whereas GT1P32 clusters with *P. migulae*, consistent with its affiliation with this species. Cryosphere-derived *Pseudomonas* strains are indicated by a blue background and snowflake symbols. Strains selected for proteomic analyses are shown in red.

High-quality genomes were obtained for all isolates (Table S3). Genome sizes ranged from 5.30 to 7.52 Mb, with G + C contents of 58.3–59.7 mol%. CheckM2 estimated completeness at 99.4%–100% with contamination <5%. Twenty strains yielded closed, single-contig chromosome assemblies; three strains (HLT2-19-2^T^, LB3P81^T^, LB1P83^T^) remained as two-contig, high-quality drafts despite hybrid assembly attempts. Genome-scale comparisons to type strains supported species-level distinctness for the 22 proposed novel species, whereas GT1P32 represented a borderline case. Strain GT1P32 showed 96.65% ANI and 69.0% dDDH to *Pseudomonas migulae* CFML 95-321^T^, placing it near the species boundary and supporting its affiliation with *P. migulae*. In contrast, the remaining 22 strains showed ANI values of 85.24%–95.71% and dDDH values of 29.0%–64.6% relative to their closest type strains (Fig. 1), all below the generally accepted species thresholds (ANI 96%; dDDH ~70%) [42]. Together with the phylogenomic and phenotypic evidence, these results support the recognition of the 22 strains as distinct novel species. Phylogenomic inference based on concatenated core genes placed all isolates within the *P. mandelii* subgroup (Fig. 2) [40, 41], the 22 candidate novel species each formed a well-supported lineage relative to currently named species, whereas GT1P32 clustered with *P. migulae*.

To place these species in a broader cryospheric context, we queried the Global Glacier Genome and Gene Database (4GDB) [43]. Best-hit ANI values were generally low (median 81.11%), with only five isolates showing high similarity (>95%) to unnamed cryospheric genomes from Inexpressible Island and Muztag Glacier, indicating that near-conspecific representation of these lineages is currently limited in available cryospheric genome catalogues. The presence of high-ANI matches to geographically distant glacier genomes further suggests that some lineages may have broad cryospheric distributions.

### Broad environmental tolerance and shared chemotaxonomic patterns

Phenotypic characterization revealed a broadly conserved *Pseudomonas*-like profile across the collection, including rod-shaped morphology, polar flagellar motility (Fig. S2), oxidase and catalase activity, and nitrate reduction (Tables S4 and S5). The strains showed broad environmental tolerance: all grew at −2°C, with strain-dependent upper growth limits ranging from 30°C to 37°C, and most tolerated approximately pH 5.0–11.0 and 3.5%–5.0% NaCl. Cellular fatty-acid profiles were dominated by C_16:0_ and unsaturated fatty acids, including summed feature 3 and summed feature 8, with strain-specific variation in C_17:0_ cyclo and C_17:1_  *ω*5*c* (Table S6). Although many traits were shared across the collection, as expected for closely related members of the *P. mandelii* subgroup, the phenotypic and chemotaxonomic data provide descriptive support for the proposed taxa and complement the genome-based species delineation.

### Nitrogen cycling potential across glacier-derived species

**Figure 3:**
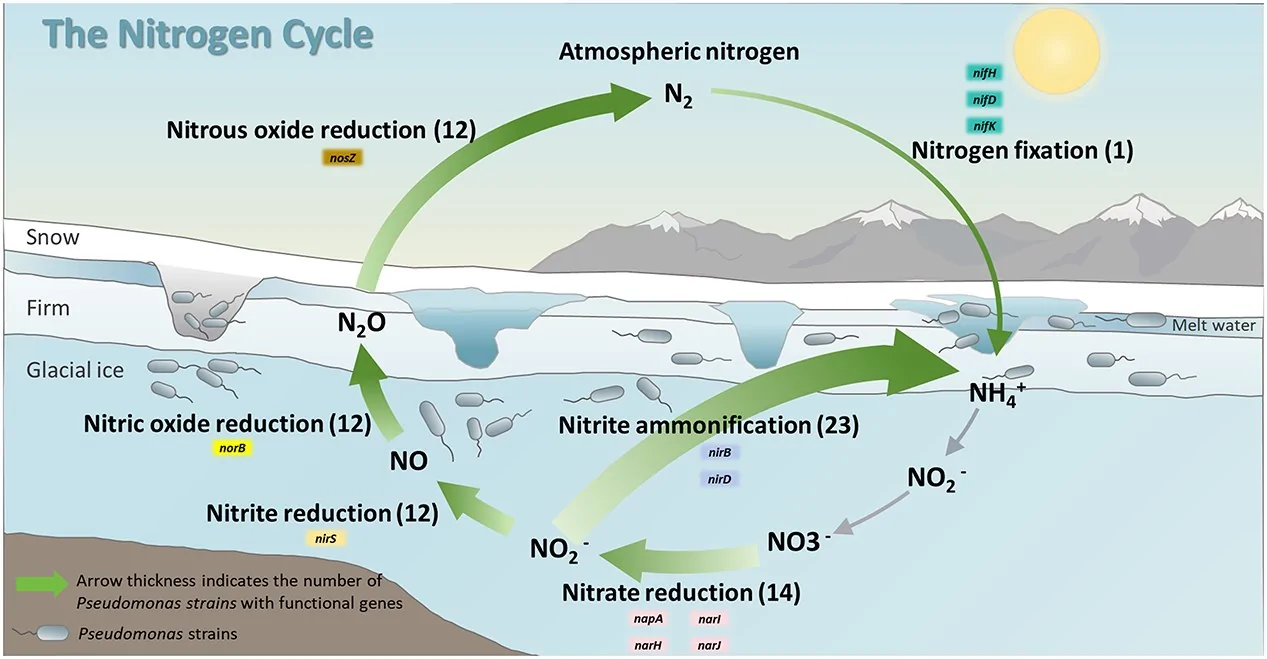
Predicted nitrogen transformation potential across 23 glacier-derived *Pseudomonas* strains. Nitrogen-transformation steps were inferred from genome-based marker genes detected in the 23 isolates. Green arrows indicate predicted transformations, with arrow thickness proportional to the number of strains encoding marker genes for each step. Numbers in parentheses indicate genome-predicted strain counts rather than phenotypic outcomes. Marker genes shown include *napA*, *narI*, *narH*, and *narJ* for nitrate reduction; *nirB* and *nirD* for nitrite ammonification; *nirS* for nitrite reduction; *norB* for nitric oxide reduction; *nosZ* for nitrous oxide reduction; and *nifH*, *nifD*, and *nifK* for nitrogen fixation.

Genome-based metabolic reconstruction revealed heterogeneous nitrogen-transformation potential across the 23 strains (Fig. 3). DNRA-related genes were detected in all strains, nitrate-reduction markers in 14 strains, complete denitrification marker sets in 12 strains, and nitrogen-fixation genes only in XS1P51^T^. These numbers represent genome-predicted potential rather than phenotypic outcomes. In contrast, phenotypic assays showed that all 23 strains reduced nitrate to nitrite, while seven strains reduced nitrate beyond nitrite under the API 20NE assay conditions. This genome–phenotype mismatch identifies nitrate reduction as a key functional trait requiring cultivation-based validation.

### Resistance- and virulence-associated genomic features

Screening against AMR and virulence-associated databases provided little evidence for acquired resistance determinants. ResFinder, ARG-ANNOT, and PlasmidFinder detected no acquired AMR genes, and NCBI AMR returned only a single *β*-lactamase hit in one strain. CARD and MEGARes mainly identified intrinsic efflux/regulatory determinants, whereas VFDB and Victors matches were dominated by motility/chemotaxis, iron uptake, and biofilm-associated functions (Table S7). Together, these results suggest limited signatures of acquired resistance and indicate that most annotated AMR/VFG-like features likely reflect environmental fitness traits rather than direct indicators of clinical risk.

### Conserved temperature-structured proteome responses and network architecture

**Figure 4:**
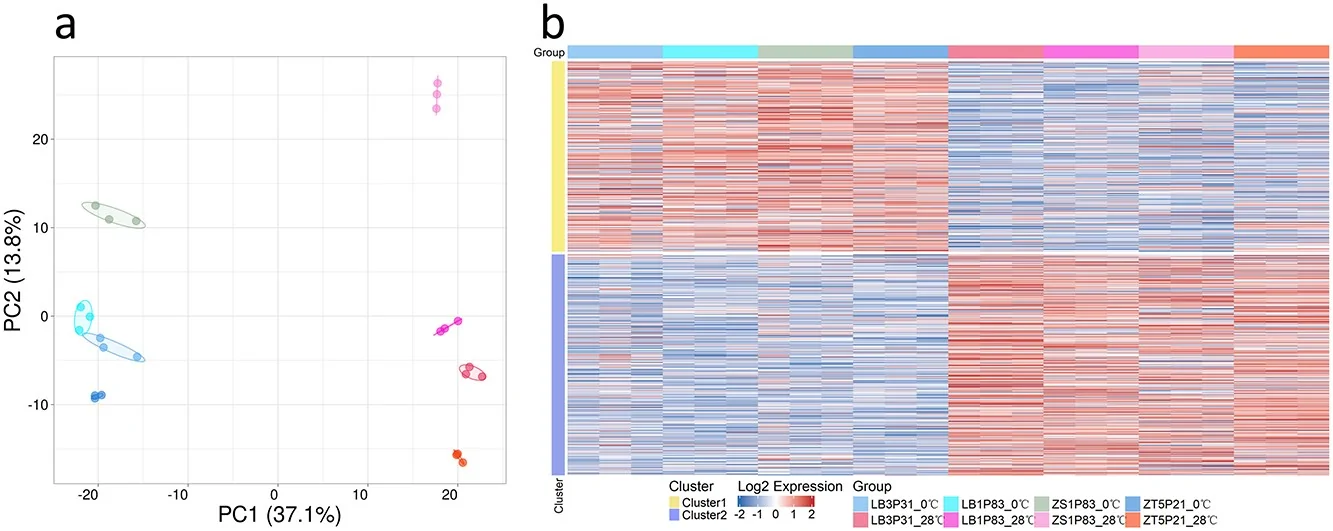
Temperature is a major determinant of proteome-wide variation across representative glacier *Pseudomonas* species. (a) PCA of quantitative proteomes, showing separation of 0°C and 28°C samples primarily along PC1. (b) Heatmap of significantly variable proteins, revealing two temperature-associated abundance modules that are broadly conserved across strains.

To identify candidate mechanisms supporting thermal acclimation, we compared proteomes of four representative species selected from distinct phylogenetic branches (Fig. 2) grown at 0°C versus 28°C. Principal component analysis (PCA) showed tight clustering of biological replicates and separation of 0°C and 28°C samples along PC1, which explained 37.1% of total variance (Fig. 4a). One-way analysis of variance (ANOVA) identified 903 proteins that varied significantly across the eight strain/temperature groups (*P* < .05). Clustering resolved two dominant temperature-associated expression modules: 418 proteins elevated at 0°C and 485 elevated at 28°C (Fig. 4b).

**Figure 5:**
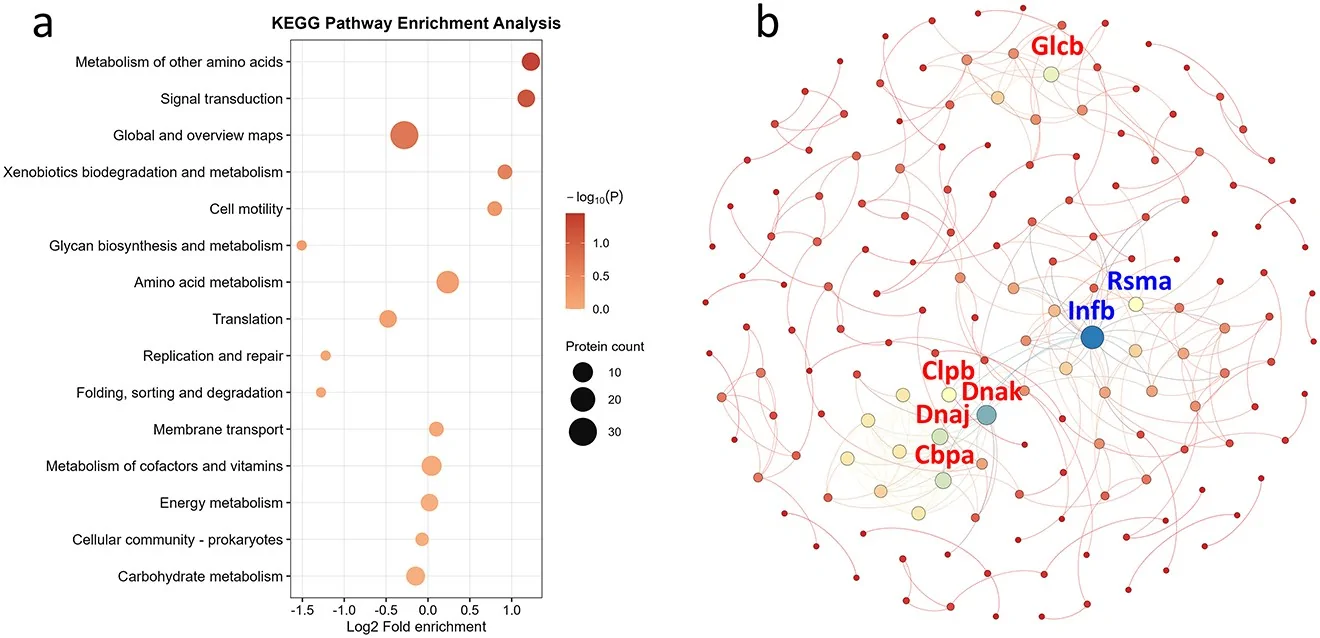
Temperature-responsive functional shifts and a shared protein association backbone across glacier-derived *Pseudomonas* species. (a) KEGG level-2 over-representation analysis of temperature-responsive proteins. Proteins showing a significant temperature main effect (*P* < .05) and an absolute abundance change of ≥3-fold (|log2FC| ≥ log2(3)) were used as the foreground set. KEGG level-2 categories were tested by Fisher’s exact test against the background of all quantified proteins with KEGG annotations. The *x*-axis shows log2 fold enrichment (observed/expected), where positive values indicate over-representation of a category among temperature-responsive proteins relative to the background, and negative values indicate under-representation. Bubble size denotes the number of foreground proteins mapped to each category. (b) Conserved protein association network across species. A merged STRING functional-association network was generated by combining strain-specific protein–protein association networks after unifying nodes by gene name. Edges were retained when supported by at least three of the four strains. The seven most central proteins are highlighted. Label colors indicate direction of temperature response in the corresponding strain-specific proteomes (red, higher at 28°C; blue, higher at 0°C).

Applying a stringent cross-strain filter (≥3-fold; *P* < .05; concordant in at least three of four strains) identified 19 conserved temperature-responsive proteins (Table S8). Eleven were more abundant at 28°C, including proteostasis factors (TapB, ClpB, MucD), redox/electron transfer proteins (Qor and azurin-like proteins), HemN, and central carbon metabolism enzymes (AcnA and GlcB), and the RNA-modification protein TtcA. Eight were elevated at 0°C, including GbcA, one-carbon/folate-linked enzymes (FdhA, GlyA3, MetE), and proteins linked to RNA processing and translation (RluB, Era, RnhB, RplL, YdiU). These proteins were further grouped into functional modules to summarize conserved temperature-responsive processes across the four representative species (Fig. S3). Proteins with higher abundance at 0°C were mainly associated with compatible-solute metabolism, one-carbon metabolism, amino-acid metabolism, and RNA/translation-related processes, whereas proteins with higher abundance at 28°C were enriched in proteostasis, redox/electron transfer, heme metabolism, and central carbon metabolism. This module-level analysis indicates that the conserved temperature response was not randomly distributed across the proteome, but was concentrated in cellular processes linked to osmotic and metabolic adjustment, protein quality control, redox balance, and carbon-flow reorganization. KEGG mapping of proteins with a significant temperature main effect (*P* < .05) and ≥ 3-fold change showed a strong metabolic bias, dominated by global/overview metabolism (18 proteins) and amino acid metabolism (12 proteins), with smaller contributions from energy, carbohydrate, and cofactor/vitamin metabolism (4 proteins each), and only limited signal transduction (4) and membrane transport (2) (Fig. S4). KEGG annotations of the temperature-responsive proteins were skewed toward metabolic functions, with the strongest nominal signal in “Metabolism of other amino acids”, although no KEGG level-2 category remained significant after Benjamini–Hochberg correction (Fig. 5a).

Protein association networks (STRING-based) unified by gene name yielded a shared functional-association backbone of 291 edges supported by at least three strains, including 84 edges shared by all four strains (Fig. 5b). The top hubs clustered into three coherent modules linked to translation initiation and post-transcriptional regulation, chaperone-mediated proteostasis, and central carbon metabolism. Several hubs also showed consistent temperature dependence across species: GlcB, ClpB, DnaJ, DnaK, and CbpA were more abundant at 28°C, whereas InfB and RsmA increased at 0°C.

## Discussion

This study provides a cultivation-anchored, genome-resolved view of glacier-associated *Pseudomonas* diversity and function across ten glaciers in western China. Core-genome phylogenomics and genome-wide relatedness metrics demonstrated that 22 isolates represent distinct novel species within the *P. mandelii* subgroup. This genome-based framework establishes a coherent evolutionary context for interpreting functional repertoires and adaptation strategies in cryospheric *Pseudomonas* lineages.

Comparisons with the 4GDB revealed generally low ANI values to existing metagenome-assembled genomes, indicating that current catalogs undersample the true species diversity of glacier *Pseudomonas*. However, a few isolates showed high ANI matches to genomes from geographically distant glaciers (e.g. Inexpressible Island and Muztag Glacier) [43], suggesting that some of these novel lineages may have broad, even global, distributions within the cryosphere. These patterns collectively support an ecology-oriented interpretation: cultivation-based and genome-informed taxonomic studies not only expand the named diversity but also provide the necessary framework for more effectively integrating species- and strain-level diversity analyses into research on the cryosphere and related ecosystems.

Phenotypic breadth across the collection—including growth at −2°C, strain-dependent upper growth limits of 30°C–37°C, growth over an approximate pH range of 5.0–11.0, and variable salt tolerance—supports the capacity of these species to persist across dynamic glacier microhabitats and potentially beyond the ice margin. In the cryosphere, micro-scale environmental heterogeneity (nutrients, meltwater chemistry, oxygen availability) can promote coexistence and diversification even among closely related taxa [4, 44]. The recovery of related lineages across geographically separated glaciers is consistent with regional-scale connectivity, potentially mediated by long-distance transport and deposition [45]. These processes are increasingly relevant under rapid deglaciation, which can enhance dispersal and establishment opportunities in forefields and downstream networks [6].

A central ecological outcome of our genome-enabled survey and phenotypic analyses is the prevalence and heterogeneity of nitrogen-transformation potential. DNRA conserves nitrogen as ammonium and may therefore support productivity in oligotrophic supraglacial and proglacial environments where nitrogen frequently constrains microbial growth [46, 47]. By contrast, nitrogen fixation appeared to be rare in our strain set, with *nifHDK* detected only in strain XS1P51^T^. This pattern is consistent with the broader literature, in which diazotrophic *Pseudomonas* have been reported only in a limited number of lineages, including *Pseudomonas stutzeri*-related strains such as *P. stutzeri* A1501 and *Pseudomonas azotifigens* [48, 49]. The rarity of this trait may reflect the high energetic cost of nitrogenase under cold, nutrient-limited conditions [50], suggesting that most glacier-derived *Pseudomonas* may depend more on flexible processing of oxidized nitrogen intermediates than on widespread atmospheric nitrogen fixation.

Consistent with this idea, all strains reduced nitrate phenotypically, despite canonical nitrate-reduction/denitrification markers being detected in only a subset of genomes. This observation highlights a practical limitation of genome-based functional inference in non-model extremophiles. Divergent or non-canonical reductases, alternative gene organizations, and annotation blind spots may lead to under-detection of nitrogen-transformation genes. In addition, the coverage of reference databases and the criteria used for marker-gene recognition may influence the recovery of denitrification-related genes, thereby contributing to the discrepancy between genome-based predictions and cultivation-based phenotypes. We did not detect consistent proteomic signals corresponding to canonical denitrification enzymes, nor were clear temperature-dependent differences observed for these enzymes between 0°C and 28°C. This likely reflects the design of the proteomic experiment, which was intended to assess general temperature-responsive physiology rather than to induce denitrification; denitrification enzyme expression is typically regulated by oxygen availability, nitrate/nitrite availability, redox status and growth phase. Importantly, this mismatch is not merely technical: it underscores why cultivated reference strains remain essential for validating biogeochemical potential and for refining marker sets and annotation pipelines for cryospheric lineages.

Notably, we found little evidence of acquired AMR determinants across the 23 genomes. This pattern is ecologically informative and consistent with limited anthropogenic influence in these remote glacial environments. Instead, the AMR- and virulence-annotated database matches detected in these genomes likely reflect conserved environmental-fitness traits, including efflux-mediated detoxification, motility/chemotaxis, iron acquisition, and biofilm-associated persistence, that support survival and competition in cryospheric niches [51]. Moreover, given their broad thermal tolerance, some lineages may persist beyond the ice margin and contribute to downstream community assembly as glacier-linked ecosystems emerge, although broader sampling will be needed to determine how often dispersal translates into establishment.

Proteomics suggests a broadly similar, temperature-structured response across the four representative species, despite differences in genetic background. The centrality of InfB and DnaK within the conserved association backbone highlights that translation control and proteostasis may play important roles in thermal acclimation, consistent with the importance of protein synthesis and folding quality control during thermal shifts. GlcB represents a major metabolic hub within this conserved architecture, and its higher abundance at 28°C is consistent with temperature-dependent engagement of the glyoxylate shunt and carbon-efficient metabolic routing under changing energetic demands [15]. Conversely, increased abundance of InfB and RsmA at 0°C points to strengthened investment in translation initiation and post-transcriptional regulation during near-freezing growth.

Extending this network-level view, the conserved temperature-responsive protein set can be resolved into several mechanistic axes. First, the higher abundance of compatible-solute- and one-carbon-metabolism proteins at 0°C, including GbcA, FdhA, GlyA3, and MetE, is consistent with increased osmoprotective and folate-linked metabolic demands during near-freezing growth. Such processes may help maintain cellular homeostasis when low temperature restricts enzyme kinetics, membrane dynamics and nutrient flux. Second, RNA- and translation-associated proteins, including Era, RnhB, RluB, RplL, and YdiU, indicate that post-transcriptional control, ribosome function, and RNA processing are important components of the low-temperature response. Third, proteins more abundant at 28°C, including ClpB, MucD, TapB, Qor, azurin-like proteins, AcnA, and GlcB, point to increased investment in proteostasis, redox/electron-transfer capacity and central carbon metabolism under warmer conditions. Together, these patterns suggest that the conserved response involves coordinated adjustment of osmotic/metabolic homeostasis at near-freezing temperature and enhanced protein-quality control and carbon-flow reorganization at 28°C.

The recurrence of these hubs and conserved edges across distinct species is consistent with a shared acclimation architecture that has been maintained across lineages, although the underlying evolutionary basis (e.g. ancestry versus convergent retention) remains to be resolved. This pattern supports the hypothesis that members of the *P. mandelii* subgroup constitute a cold-adapted ecological assemblage that deploys conserved molecular modules to cope with both sustained low temperatures and episodic warming. By emphasizing conserved network architecture rather than individual proteins, we outline a mechanism-based, testable framework: lineages retaining this toolkit may be better equipped to tolerate thermal fluctuations and therefore may have enhanced potential to persist along the ice–forefield continuum as deglaciation proceeds. Accordingly, network-level conservation provides testable predictions about how these lineages could respond to a changing cryosphere.

## Conclusion

By integrating cultivation-based isolation, high-quality genome assemblies, phenotypic characterization, functional genomics, and comparative proteomics, our study moves beyond descriptive surveys to deliver a mechanistic, species-level resolution of *Pseudomonas* diversification in glacier ecosystems. The 22 novel species proposed here, together with one isolate affiliated with *P. migulae*, expand cultured references for investigating how cryospheric bacteria function across the ice–forefield transition. These lineages provide valuable benchmarks for assessing the impacts of deglaciation-driven dispersal on nitrogen cycling in rapidly warming environments. On the basis of polyphasic taxonomic evidence, we formally propose the following 22 novel species within the genus *Pseudomonas* (Table S9).

### Description of *Pseudomonas trehalosi* sp. nov.

*Pseudomonas trehalosi* (tre.ha.lo’si. N.L. gen. n. *trehalosi*, of trehalose).

Cells are Gram-stain-negative, aerobic, motile by means of a monopolar flagellum, 0.9–2.1 μm in length and 0.9–1.6 μm in width. Colonies are round and pale yellow after 1–2 days of incubation at 28°C on Nutrient Agar plate. Growth occurs at −2°C to +30°C (optimum, 25°C–28°C), pH 5.0–11.0 (optimum, pH 7.0), and 0%–5.0% (w/v) NaCl (optimum, 1%). Oxidase- and catalase-positive. Hydrolyses casein, gelatin, starch, and tributyrin, but not esculin and Tween 80. Positive for reduction of nitrates to nitrites, citrate utilization, Voges–Proskauer test. Negative for H_2_S production, indole production, fermentation of glucose. Produces acid from L-rhamnose, melibiose, and L-arabinose. Assimilates glycerol, L-arabinose, D-ribose, D-galactose, D-glucose, D-fructose, D-mannose, inositol, D-mannitol, D-sorbitol, trehalose, D-lyxose, D-arabitol, and gluconate. The major cellular fatty acids are C_16:0_, summed feature 3 (C_16:1_  *ω*7*c* and/or C_16:1_  *ω*6*c*), and summed feature 8 (C_18:1_  *ω*6*c*/*ω*7*c*). The genomic DNA G + C content is 58.6 mol%.

The type strain Hz4^T^ (= CGMCC 1.9264^T^ = JCM 37121^T^) was isolated from a cryoconite sample collected from the Xinjiang No.1 Glacier, P.R. China. The GenBank accession number for the 16S rRNA gene sequence is JX949205. The genome sequence has been deposited at DDBJ/ENA/GenBank under the accession number JBUBVP000000000.

### Description of *Pseudomonas raffinosi* sp. nov

*Pseudomonas raffinosi* (raf.fi.no’si. N.L. gen. n. *raffinosi*, of raffinose).

Cells are Gram-stain-negative, aerobic, motile by means of a monopolar flagellum, 1.1–2.4 μm in length and 0.8–1.0 μm in width. Colonies are round and pale yellow after 1–2 days of incubation at 28°C on Nutrient Agar plate. Growth occurs at −2°C to +32°C (optimum, 25°C–30°C), pH 5.0–11.0 (optimum, pH 7.0), and 0%–5.0% (w/v) NaCl (optimum, 1%). Oxidase- and catalase-positive. Hydrolyses casein, starch, and tributyrin, but not gelatin, esculin and Tween 80. Positive for reduction of nitrates to nitrites, citrate utilization, Voges–Proskauer test, fermentation of glucose. Negative for H_2_S production, indole production. Produces acid from D-glucose, D-mannitol, L-rhamnose, melibiose, amygdalin, and L-arabinose. Assimilates glycerol, L-arabinose, D-ribose, D-xylose, D-galactose, D-glucose, D-fructose, D-mannose, D-mannitol, raffinose, D-arabitol, gluconate, and 2-ketogluconate. The major cellular fatty acids are C_16:0_, summed feature 3 (C_16:1_  *ω*7*c* and/or C_16:1_  *ω*6*c*), C_17:0_ cyclo, and summed feature 8 (C_18:1_  *ω*6*c*/*ω*7*c*). The genomic DNA G + C content is 58.3 mol%.

The type strain TMB3-21^T^ (= CGMCC 1.9707^T^ = JCM 37139^T^) was isolated from an ice sample collected from the Toumingmengke Glacier located in Gansu Province, P.R. China. The GenBank accession number for the 16S rRNA gene sequence is JX949981. The genome sequence has been deposited at DDBJ/ENA/GenBank under the accession number JBUBVO000000000.

### Description of *Pseudomonas algoris* sp. nov

*Pseudomonas algoris* (al’go.ris. L. gen. n. *algoris*, of the cold).

Cells are Gram-stain-negative, aerobic, motile by means of a monopolar flagellum, 1.3–3.8 μm in length and 0.5–1.1 μm in width. Colonies are 1–3 mm in diameter, round and pale yellow after 1–2 days of incubation at 28°C on Nutrient Agar plate. Growth occurs at −2°C to +37°C (optimum, 25°C–30°C), pH 6.0–11.0 (optimum, pH 7.0), and 0%–4.0% (w/v) NaCl (optimum, 1%). Oxidase- and catalase-positive. Hydrolyses casein, gelatin, starch, and tributyrin, but not esculin and Tween 80. Positive for reduction of nitrates to nitrites, citrate utilization, Voges–Proskauer test. Negative for H_2_S production, indole production, fermentation of glucose. Produces acid from sorbitol, L-rhamnose, melibiose, and L-arabinose. Assimilates glycerol, L-arabinose, D-ribose, D-galactose, D-glucose, D-fructose, D-mannose, inositol, D-mannitol, D-sorbitol, N-acetylglucosamine, trehalose, D-lyxose, D-arabitol, and gluconate. The major cellular fatty acids are C_16:0_, summed feature 3 (C_16:1_  *ω*7*c* and/or C_16:1_  *ω*6*c*), and summed feature 8 (C_18:1_  *ω*6*c*/*ω*7*c*). The genomic DNA G + C content is 58.6 mol%.

The type strain MDT1-16^T^ (= CGMCC 1.9794^T^ = JCM 37132^T^) was isolated from a cryoconite sample collected from the Midui Glacier located in Tibet Autonomous Region, P.R. China. The GenBank accession number for the 16S rRNA gene sequence is JX949552. The genome sequence has been deposited at DDBJ/ENA/GenBank under the accession number JBUBVN000000000.

### Description of *Pseudomonas facultativa* sp. nov

*Pseudomonas facultativa* (fa.cul.ta.ti’va. L. fem. n. *facultas*, possibility, opportunity; N.L. fem. adj. *facultativa*, facultative, referring to facultatively anaerobic growth).

Cells are Gram-stain-negative, facultatively anaerobic, motile by means of a monopolar flagellum, 1.1–3.1 μm in length and 0.8–1.1 μm in width. Colonies are round and pale yellow after 1–2 days of incubation at 28°C on Nutrient Agar plate. Growth occurs at −2°C to +30°C (optimum, 25°C–28°C), pH 5.0–11.0 (optimum, pH 7.0), and 0%–5.0% (w/v) NaCl (optimum, 1%). Oxidase- and catalase-positive. Hydrolyses gelatin and tributyrin, but not casein, starch, esculin, and Tween 80. Positive for reduction of nitrates to nitrites, citrate utilization, Voges–Proskauer test. Negative for H_2_S production, indole production, fermentation of glucose. Produces acid from L-rhamnose, melibiose, amygdalin, L-arabinose. Assimilates glycerol, L-arabinose, D-ribose, D-galactose, D-glucose, D-fructose, D-mannose, inositol, D-mannitol, N-acetylglucosamine, D-lyxose, D-arabitol, and gluconate. The major cellular fatty acids are C_16:0_, summed feature 3 (C_16:1_  *ω*7*c* and/or C_16:1_  *ω*6*c*), summed feature 8 (C_18:1_  *ω*6*c*/*ω*7*c*), and C_17:0_ cyclo. The genomic DNA G + C content is 58.5 mol%.

The type strain MDT1-17^T^ (= CGMCC 1.9795^T^ = JCM 37133^T^) was isolated from a cryoconite sample collected from the Midui Glacier located in Tibet Autonomous Region, P.R. China. The GenBank accession number for the 16S rRNA gene sequence is JX949553. The genome sequence has been deposited at DDBJ/ENA/GenBank under the accession number JBUBVM000000000.

### Description of *Pseudomonas glaciei* sp. nov

*Pseudomonas glaciei* (gla.ci.e’i. N.L. gen. n. *glaciei*, of ice, of a glacier).

Cells are Gram-stain-negative, facultatively anaerobic, motile by means of a monopolar flagellum, 1.2–2.2 μm in length and 0.8–1.2 μm in width. Colonies are round and pale yellow after 1–2 days of incubation at 28°C on Nutrient Agar plate. Growth occurs at −2°C to +32°C (optimum, 25°C to 30°C), pH 5.0–11.0 (optimum, pH 7.0), and 0%–4.0% (w/v) NaCl (optimum, 1%). Oxidase- and catalase-positive. Hydrolyses casein, gelatin, and tributyrin, but not starch, esculin, and Tween 80. Positive for reduction of nitrates to nitrites, citrate utilization, Voges–Proskauer test. Negative for H_2_S production, indole production, fermentation of glucose. Produces acid from melibiose and L-arabinose. Assimilates glycerol, L-arabinose, D-ribose, D-galactose, D-glucose, D-fructose, D-mannose, inositol, D-mannitol, N-acetylglucosamine, trehalose, D-arabitol, and gluconate. The major cellular fatty acids are C_16:0_, C_17:0_ cyclo, summed feature 8 (C_18:1_  *ω*6*c*/*ω*7*c*), and summed feature 3 (C_16:1_  *ω*7*c* and/or C_16:1_  *ω*6*c*). The genomic DNA G + C content is 58.8 mol%.

The type strain MDT2-39-1^T^ (= CGMCC 1.9900^T^ = JCM 37135^T^) was isolated from a cryoconite sample collected from the Midui Glacier located in Tibet Autonomous Region, P.R. China. The GenBank accession number for the 16S rRNA gene sequence is JX949558. The genome sequence has been deposited at DDBJ/ENA/GenBank under the accession number JBUBVL000000000.

### Description of *Pseudomonas kochi* sp. nov

*Pseudomonas kochi* (ko’chi. N.L. gen. n. *kochi*, of Robert Koch).

Cells are Gram-stain-negative, aerobic, motile by means of a monopolar flagellum, 1.3–2.4 μm in length and 0.8–1.1 μm in width. Colonies are round and pale yellow after 1–2 days of incubation at 28°C on Nutrient Agar plate. Growth occurs at −2°C to +32°C (optimum, 25°C–30°C), pH 6.0–11.0 (optimum, pH 7.0), and 0%–4.0% (w/v) NaCl (optimum, 1%). Oxidase- and catalase-positive. Hydrolyses casein, gelatin, and tributyrin, but not starch, esculin, and Tween 80. Positive for reduction of nitrates to nitrites, citrate utilization, Voges–Proskauer test. Negative for H_2_S production, indole production, fermentation of glucose. Produces acid from D-glucose, L-rhamnose, sucrose, melibiose, amygdalin, and L-arabinose. Assimilates glycerol, L-arabinose, D-ribose, D-galactose, D-glucose, D-fructose, D-mannose, inositol, D-mannitol, N-acetylglucosamine, sucrose, trehalose, D-arabitol, and gluconate. The major cellular fatty acids are summed feature 3 (C_16:1_  *ω*7*c* and/or C_16:1_  *ω*6*c*), C_16:0_, and summed feature 8 (C_18:1_  *ω*6*c*/*ω*7*c*). The genomic DNA G + C content is 58.6 mol%.

The type strain MDT1-85^T^ (= CGMCC 1.9936^T^ = JCM 37134^T^) was isolated from a cryoconite sample collected from the Midui Glacier located in Tibet Autonomous Region, P.R. China. The GenBank accession number for the 16S rRNA gene sequence is JX949570. The genome sequence has been deposited at DDBJ/ENA/GenBank under the accession number JBUBVK000000000.

### Description of *Pseudomonas brevis* sp. nov

*Pseudomonas brevis* (bre’vis. L. fem. adj. *brevis*, short).

Cells are Gram-stain-negative, aerobic, motile by means of a monopolar flagellum, 1.0–2.0 μm in length and 0.9–1.1 μm in width. Colonies are round and pale yellow after 1–2 days of incubation at 28°C on Nutrient Agar plate. Growth occurs at −2°C to +30°C (optimum, 25°C–28°C), pH 5.0–11.0 (optimum, pH 7.0), and 0%–4.0% (w/v) NaCl (optimum, 1%). Oxidase- and catalase-positive. Hydrolyses casein, gelatin, starch, and tributyrin, but not esculin and Tween 80. Positive for nitrate reduction beyond nitrite, citrate utilization, Voges–Proskauer test, fermentation of glucose. Negative for H_2_S production, indole production. Produces acid from melibiose and L-arabinose. Assimilates glycerol, D-ribose, D-glucose, D-fructose, D-mannose, inositol, D-mannitol, D-sorbitol, N-acetylglucosamine, trehalose, D-arabitol, and gluconate. The major cellular fatty acids are summed feature 3 (C_16:1_  *ω*7*c* and/or C_16:1_  *ω*6*c*), C_16:0_, and summed feature 8 (C_18:1_  *ω*6*c*/*ω*7*c*). The genomic DNA G + C content is 58.4 mol%.

The type strain HLT2–19-2^T^ (= CGMCC 1.10010^T^ = JCM 37120^T^) was isolated from a cryoconite sample collected from the Hailuogou Glacier located in Sichuan Province, P.R. China. The GenBank accession number for the 16S rRNA gene sequence is JX949408. The genome sequence has been deposited at DDBJ/ENA/GenBank under the accession number JBUBVJ000000000.

### Description of *Pseudomonas woesei* sp. nov

*Pseudomonas woesei* (woe’se.i. N.L. gen. n. *woesei*, of Woese, referring to Carl R. Woese).

Cells are Gram-stain-negative, aerobic, motile by means of a monopolar flagellum, 1.2–2.5 μm in length and 0.9–1.2 μm in width. Colonies are round and pale yellow after 1–2 days of incubation at 28°C on Nutrient Agar plate. Growth occurs at −2°C to +32°C (optimum, 25°C–30°C), pH 5.0–11.0 (optimum, pH 7.0), and 0%–4.0% (w/v) NaCl (optimum, 1%). Oxidase- and catalase-positive. Hydrolyses casein, gelatin, and tributyrin, but not starch, esculin and Tween 80. Positive for reduction of nitrates to nitrites, citrate utilization. Negative for H_2_S production, indole production, Voges–Proskauer test, fermentation of glucose. Produces acid from L-rhamnose, melibiose, and L-arabinose. Assimilates glycerol, L-arabinose, D-galactose, D-glucose, D-fructose, D-mannose, inositol, D-mannitol, N-acetylglucosamine, trehalose, D-arabitol, gluconate, and 2-ketogluconate. The major cellular fatty acids are C_16:0_, C_17:1_  *ω*5*c*, summed feature 3 (C_16:1_  *ω*7*c* and/or C_16:1_  *ω*6*c*), and summed feature 8 (C_18:1_  *ω*6*c*/*ω*7*c*). The genomic DNA G + C content is 59.1 mol%.

The type strain LB3P93^T^ (= CGMCC 1.11279^T^ = JCM 37129^T^) was isolated from an ice sample collected from the Laigu Glacier located in Tibet Autonomous Region, P.R. China. The GenBank accession number for the 16S rRNA gene sequence is PX415443. The genome sequence has been deposited at DDBJ/ENA/GenBank under the accession number JBUBVI000000000.

### Description of *Pseudomonas salicini* sp. nov

*Pseudomonas salicini* (sa.li.ci’ni. N.L. gen. n. *salicini*, of salicin).

Cells are Gram-stain-negative, aerobic, motile by means of a monopolar flagellum, 1.0–2.4 μm in length and 0.8–1.1 μm in width. Colonies are round and pale yellow after 1–2 days of incubation at 28°C on Nutrient Agar plate. Growth occurs at −2°C to +32°C (optimum, 25°C–30°C), pH 5.0–11.0 (optimum, pH 7.0), and 0%–5.0% (w/v) NaCl (optimum, 1%). Oxidase- and catalase-positive. Hydrolyses casein, gelatin, starch, tributyrin, and esculin, but not Tween 80. Positive for reduction of nitrates to nitrites, citrate utilization, Voges–Proskauer test. Negative for H_2_S production, indole production, fermentation of glucose. Produces acid from L-rhamnose, sucrose, melibiose, amygdalin, and L-arabinose. Assimilates glycerol, L-arabinose, D-ribose, D-galactose, D-glucose, D-fructose, D-mannose, inositol, D-mannitol, D-sorbitol, N-acetylglucosamine, esculin, salicin, sucrose, trehalose, D-arabitol, gluconate, and 2-ketogluconate. The major cellular fatty acids are C_16:0_ and C_17:0_ cyclo. The genomic DNA G + C content is 58.7 mol%.

The type strain LB3P25^T^ (= CGMCC 1.11286^T^ = JCM 37124^T^) was isolated from an ice sample collected from the Laigu Glacier located in Tibet Autonomous Region, P.R. China. The GenBank accession number for the 16S rRNA gene sequence is PX415444. The genome sequence has been deposited at DDBJ/ENA/GenBank under the accession number JBUBVH000000000.

### Description of *Pseudomonas cellobiosi* sp. nov

*Pseudomonas cellobiosi* (cel.lo.bi.o’si. N.L. gen. n. *cellobiosi*, of cellobiose).

Cells are Gram-stain-negative, aerobic, motile by means of a monopolar flagellum, 1.1–2.4 μm in length and 0.9–1.2 μm in width. Colonies are round and pale yellow after 1–2 days of incubation at 28°C on Nutrient Agar plate. Growth occurs at −2°C to +32°C (optimum, 25°C–30°C), pH 5.0–11.0 (optimum, pH 7.0), and 0%–3.5% (w/v) NaCl (optimum, 1%). Oxidase- and catalase-positive. Hydrolyses tributyrin, but not casein, gelatin, starch, esculin, and Tween 80. Positive for reduction of nitrates to nitrites, citrate utilization, Voges–Proskauer test. Negative for H_2_S production, indole production, fermentation of glucose. Assimilates glycerol, D-ribose, D-glucose, D-fructose, D-mannose, D-mannitol, D-sorbitol, methyl-α-D-glucopyranoside, esculin, cellobiose, maltose, sucrose, trehalose, glycogen, gentiobiose, and gluconate. The major cellular fatty acids are C_16:0_, C_17:1_  *ω*5*c*, summed feature 8 (C_18:1_  *ω*6*c*/*ω*7*c*), and summed feature 3 (C_16:1_  *ω*7*c* and/or C_16:1_  *ω*6*c*). The genomic DNA G + C content is 59.0 mol%.

The type strain LB3P81^T^ (= CGMCC 1.11292^T^ = JCM 37128^T^) was isolated from an ice sample collected from the Laigu Glacier located in Tibet Autonomous Region, P.R. China. The GenBank accession number for the 16S rRNA gene sequence is PX415445. The genome sequence has been deposited at DDBJ/ENA/GenBank under the accession number JBUBVG000000000.

### Description of *Pseudomonas leeuwenhoeki* sp. nov

*Pseudomonas leeuwenhoeki* (lee.uwen.hoek’i. N.L. gen. n. *leeuwenhoeki*, of Antonie van Leeuwenhoek).

Cells are Gram-stain-negative, aerobic, motile by means of a monopolar flagellum, 1.0–2.0 μm in length and 0.8–1.1 μm in width. Colonies are round and pale yellow after 1–2 days of incubation at 28°C on Nutrient Agar plate. Growth occurs at −2°C to +30°C (optimum, 25°C–28°C), pH 5.0–11.0 (optimum, pH 7.0), and 0%–5.0% (w/v) NaCl (optimum, 1%). Oxidase- and catalase-positive. Hydrolyses tributyrin, but not casein, gelatin, starch, esculin, and Tween 80. Positive for reduction of nitrates to nitrites, citrate utilization, Voges–Proskauer test, fermentation of glucose. Negative for H_2_S production, indole production. Produces acid from D-glucose, L-rhamnose, melibiose, and L-arabinose. Assimilates glycerol, D-ribose, D-galactose, D-glucose, D-fructose, D-mannose, gluconate, and 2-ketogluconate. The major cellular fatty acids are C_16:0_, summed feature 3 (C_16:1_  *ω*7*c* and/or C_16:1_  *ω*6*c*), summed feature 8 (C_18:1_  *ω*6*c*/*ω*7*c*), and C_17:0_ cyclo. The genomic DNA G + C content is 59.7 mol%.

The type strain LB3P31^T^ (= CGMCC 1.11296^T^ = JCM 37125^T^) was isolated from an ice sample collected from the Laigu Glacier located in Tibet Autonomous Region, P.R. China. The GenBank accession number for the 16S rRNA gene sequence is PX415446. The genome sequence has been deposited at DDBJ/ENA/GenBank under the accession number JBUBVF000000000.

### Description of *Pseudomonas linnaei* sp. nov

*Pseudomonas linnaei* (lin.nae’i. N.L. gen. n. *linnaei*, of Carl Linnaeus).

Cells are Gram-stain-negative, facultatively anaerobic, motile by means of a monopolar flagellum, 1.0–2.2 μm in length and 0.7–1.0 μm in width. Colonies are round and pale yellow after 1–2 days of incubation at 28°C on Nutrient Agar plate. Growth occurs at −2°C to +32°C (optimum, 25°C–30°C), pH 6.0–11.0 (optimum, pH 7.0), and 0%–4.0% (w/v) NaCl (optimum, 1%). Oxidase- and catalase-positive. Hydrolyses casein, gelatin, and tributyrin, but not starch, esculin and Tween 80. Positive for nitrate reduction beyond nitrite, citrate utilization, Voges–Proskauer test. Negative for H_2_S production, indole production, fermentation of glucose. Produces acid from D-glucose, L-rhamnose, sucrose, melibiose, amygdalin, and L-arabinose. Assimilates glycerol, L-arabinose, D-ribose, D-xylose, D-galactose, D-glucose, D-fructose, D-mannose, inositol, D-mannitol, N-acetylglucosamine, sucrose, trehalose, D-arabitol, gluconate, and 2-ketogluconate. The major cellular fatty acids are C_16:0_, summed feature 3 (C_16:1_  *ω*7*c* and/or C_16:1_  *ω*6*c*), summed feature 8 (C_18:1_  *ω*6*c*/*ω*7*c*), and C_17:0_ cyclo. The genomic DNA G + C content is 59.0 mol%.

The type strain LB3P58^T^ (= CGMCC 1.11297^T^ = JCM 37127^T^) was isolated from an ice sample collected from the Laigu Glacier located in Tibet Autonomous Region, P.R. China. The GenBank accession number for the 16S rRNA gene sequence is PX415447. The genome sequence has been deposited at DDBJ/ENA/GenBank under the accession number JBUBVE000000000.

### Description of *Pseudomonas frigoriphila* sp. nov

*Pseudomonas frigoriphila* (fri.go.ri’phi.la. L. masc. n. *frigor*, the cold; N.L. masc. adj. *philus* (from Gr. masc. adj. *philos*), loving; N.L. fem. adj. *frigoriphila* cold-loving).

Cells are Gram-stain-negative, aerobic, motile by means of a monopolar flagellum, 1.5–2.5 μm in length and 1.0–1.2 μm in width. Colonies are round and pale yellow after 1–2 days of incubation at 28°C on Nutrient Agar plate. Growth occurs at −2°C to +32°C (optimum, 25°C–30°C), pH 6.0–11.0 (optimum, pH 7.0), and 0%–4.0% (w/v) NaCl (optimum, 1%). Oxidase- and catalase-positive. Hydrolyses casein, gelatin, starch, and tributyrin, but not esculin and Tween 80. Positive for reduction of nitrates to nitrites, citrate utilization, Voges–Proskauer test. Negative for H_2_S production, indole production, fermentation of glucose. Produces acid from D-glucose, L-rhamnose, melibiose, amygdalin, and L-arabinose. Assimilates glycerol, D-arabinose, L-arabinose, D-ribose, D-galactose, D-glucose, D-fructose, D-mannose, inositol, D-mannitol, D-sorbitol, trehalose, D-arabitol, gluconate, 2-ketogluconate, and 5-ketogluconate. The major cellular fatty acids are C_16:0_, summed feature 3 (C_16:1_  *ω*7*c* and/or C_16:1_  *ω*6*c*), and C_17:0_ cyclo. The genomic DNA G + C content is 58.8 mol%.

The type strain LB3P14^T^ (= CGMCC 1.11306^T^ = JCM 37123^T^) was isolated from an ice sample collected from the Laigu Glacier located in Tibet Autonomous Region, P.R. China. The GenBank accession number for the 16S rRNA gene sequence is PX415448. The genome sequence has been deposited at DDBJ/ENA/GenBank under the accession number JBUBVD000000000.

### Description of *Pseudomonas sorbitolis* sp. nov

*Pseudomonas sorbitolis* (sor.bi.to’lis. N.L. gen. n. *sorbitolis*, of sorbitol).

Cells are Gram-stain-negative, aerobic, motile by means of a monopolar flagellum, 1.3–3.1 μm in length and 1.0–1.2 μm in width. Colonies are round and pale yellow after 1–2 days of incubation at 28°C on Nutrient Agar plate. Growth occurs at −2°C to +32°C (optimum, 25°C–30°C), pH 5.0–11.0 (optimum, pH 7.0), and 0%–5.0% (w/v) NaCl (optimum, 1%). Oxidase- and catalase-positive. Hydrolyses casein, gelatin, and tributyrin, but not starch, esculin, and Tween 80. Positive for reduction of nitrates to nitrites, citrate utilization, Voges–Proskauer test. Negative for H_2_S production, indole production, fermentation of glucose. Produces acid from D-glucose, L-rhamnose, melibiose, amygdalin, and L-arabinose. Assimilates glycerol, L-arabinose, D-ribose, D-galactose, D-glucose, D-fructose, D-mannose, inositol, D-mannitol, N-acetylglucosamine, trehalose, D-arabitol, gluconate, and 2-ketogluconate. The major cellular fatty acids are C_17:1_  *ω*5*c* and summed feature 8 (C_18:1_  *ω*6*c*/*ω*7*c*). The genomic DNA G + C content is 59.0 mol%.

The type strain LB1P83^T^ (= CGMCC 1.11333^T^ = JCM 37122^T^) was isolated from an ice sample collected from the Laigu Glacier located in Tibet Autonomous Region, P.R. China. The GenBank accession number for the 16S rRNA gene sequence is PX415449. The genome sequence has been deposited at DDBJ/ENA/GenBank under the accession number JBUBVC000000000.

### Description of *Pseudomonas maltosi* sp. nov

*Pseudomonas maltosi* (mal.to’si. N.L. gen. n., *maltosi*, of maltose).

Cells are Gram-stain-negative, aerobic, motile by means of a monopolar flagellum, 1.1–1.9 μm in length and 0.8–1.1 μm in width. Colonies are round and pale yellow after 1–2 days of incubation at 28°C on Nutrient Agar plate. Growth occurs at −2°C to +32°C (optimum, 25°C–30°C), pH 5.0–11.0 (optimum, pH 7.0), and 0–%4.0% (w/v) NaCl (optimum, 1%). Oxidase- and catalase-positive. Hydrolyses casein, gelatin, esculin, and tributyrin, but not starch and Tween 80. Positive for nitrate reduction beyond nitrite, citrate utilization, Voges–Proskauer test. Negative for H_2_S production, indole production, fermentation of glucose. Produces acid from D-glucose, D-mannitol, inositol, L-rhamnose, sucrose, melibiose, amygdalin, and L-arabinose. Assimilates glycerol, L-arabinose, D-ribose, D-galactose, D-glucose, D-fructose, D-mannose, inositol, D-mannitol, N-acetylglucosamine, maltose, sucrose, trehalose, D-arabitol, gluconate, and 2-ketogluconate. The major cellular fatty acids are summed feature 3 (C_16:1_  *ω*7*c* and/or C_16:1_  *ω*6*c*), C_16:0_, and summed feature 8 (C_18:1_  *ω*6*c*/*ω*7*c*). The genomic DNA G + C content is 58.8 mol%.

The type strain LS2P72^T^ (= CGMCC 1.11413^T^ = JCM 37130^T^) was isolated from a meltwater sample collected from the Laigu Glacier located in Tibet Autonomous Region, P.R. China. The GenBank accession number for the 16S rRNA gene sequence is PX415450. The genome sequence has been deposited at DDBJ/ENA/GenBank under the accession number JBUBVB000000000.

### Description of *Pseudomonas xylosi* sp. nov

*Pseudomonas xylosi* (xy.lo’si. N.L. gen. n. *xylosi*, of xylose).

Cells are Gram-stain-negative, aerobic, motile by means of a monopolar flagellum, 1.6–3.2 μm in length and 1.0–1.1 μm in width. Colonies are round and pale yellow after 1–2 days of incubation at 28°C on Nutrient Agar plate. Growth occurs at −2°C to +32°C (optimum, 25°C–30°C), pH 5.0–11.0 (optimum, pH 7.0), and 0%–4.0% (w/v) NaCl (optimum, 1%). Oxidase- and catalase-positive. Hydrolyses gelatin and tributyrin, but not esculin, casein, starch and Tween 80. Positive for nitrate reduction beyond nitrite, citrate utilization. Negative for H_2_S production, indole production, Voges–Proskauer test, fermentation of glucose. Produces acid from D-glucose, D-mannitol, inositol, sorbitol, L-rhamnose, sucrose, melibiose, amygdalin, and L-arabinose. Assimilates glycerol, L-arabinose, D-ribose, D-xylose, D-galactose, D-glucose, D-fructose, D-mannose, inositol, D-mannitol, trehalose, D-lyxose, D-arabitol, gluconate, and 2-ketogluconate. The major cellular fatty acids are C_16:0_ and C_17:1_  *ω*5*c*. The genomic DNA G + C content is 59.3 mol%.

The type strain XS1P51^T^ (= CGMCC 1.23184^T^ = JCM 37140^T^) was isolated from a meltwater sample collected from the Zhuxi Glacier located in Tibet Autonomous Region, P.R. China. The GenBank accession number for the 16S rRNA gene sequence is PX415451. The genome sequence has been deposited at DDBJ/ENA/GenBank under the accession number JBUBVA000000000.

### Description of *Pseudomonas liquefacta* sp. nov

*Pseudomonas liquefacta* (li.que.fac’ta. L. fem. part. adj. *liquefacta*, melted, liquefied; referring to meltwater).

Cells are Gram-stain-negative, aerobic, motile by means of a monopolar flagellum, 1.2–2.2 μm in length and 0.8–1.0 μm in width. Colonies are round and pale yellow after 1–2 days of incubation at 28°C on Nutrient Agar plate. Growth occurs at −2°C to +30°C (optimum, 25°C–30°C), pH 5.0–11.0 (optimum, pH 7.0), and 0%–4.0% (w/v) NaCl (optimum, 1%). Oxidase- and catalase-positive. Hydrolyses gelatin, casein, starch, and tributyrin, but not esculin and Tween 80. Positive for nitrate reduction beyond nitrite, citrate utilization, Voges–Proskauer test. Negative for H_2_S production, indole production, fermentation of glucose. Produces acid from melibiose and L-arabinose. Assimilates glycerol, L-arabinose, D-ribose, D-galactose, D-glucose, D-fructose, D-mannose, inositol, D-mannitol, D-sorbitol, trehalose, D-lyxose, D-arabitol, and gluconate. The major cellular fatty acids are C_16:0_, summed feature 3 (C_16:1_  *ω*7*c* and/or C_16:1_  *ω*6*c*), and C_17:0_ cyclo. The genomic DNA G + C content is 58.7 mol%.

The type strain ZS1P83^T^ (= CGMCC 1.23236^T^ = JCM 37142^T^) was isolated from a meltwater sample of Zepu Glacier located in Tibet Autonomous Region, P.R. China. The GenBank accession number for the 16S rRNA gene sequence is PX415452. The genome sequence has been deposited at DDBJ/ENA/GenBank under the accession number JBUBUZ000000000.

### Description of *Pseudomonas alpina* sp. nov

*Pseudomonas alpina* (al.pi’na. L. fem. adj. *alpina*, alpine).

Cells are Gram-stain-negative, aerobic, motile by means of a monopolar flagellum, 1.4–2.4 μm in length and 0.9–1.0 μm in width. Colonies are round and pale yellow after 1–2 days of incubation at 28°C on Nutrient Agar plate. Growth occurs at −2°C to +32°C (optimum, 25°C–30°C), pH 5.0–11.0 (optimum, pH 7.0), and 0%–4.0% (w/v) NaCl (optimum, 1%). Oxidase- and catalase-positive. Hydrolyses starch and tributyrin, but not gelatin, casein, esculin, and Tween 80. Positive for reduction of nitrates to nitrites, citrate utilization, Voges–Proskauer test, fermentation of glucose. Negative for H_2_S production, urease, indole production. Produces acid from D-glucose, L-rhamnose, melibiose, and L-arabinose. Assimilates glycerol, L-arabinose, D-ribose, D-galactose, D-glucose, D-fructose, D-mannose, and gluconate. The major cellular fatty acids are C_16:0_, summed feature 3 (C_16:1_  *ω*7*c* and/or C_16:1_  *ω*6*c*), C_17:0_ cyclo, and summed feature 8 (C_18:1_  *ω*6*c*/*ω*7*c*). The genomic DNA G + C content is 58.8 mol%.

The type strain ZT5P21^T^ (= CGMCC 1.23314^T^ = JCM 37143^T^) was isolated from a cryoconite sample of Zepu Glacier located in Tibet Autonomous Region, P.R. China. The GenBank accession number for the 16S rRNA gene sequence is PX415453. The genome sequence has been deposited at DDBJ/ENA/GenBank under the accession number JBUBUY000000000.

### Description of *Pseudomonas cryoconiti* sp. nov

*Pseudomonas cryoconiti* (cry.o.co.ni’ti. N.L. neut. n. *cryoconitum*, cryoconite; N.L. gen. neut. n. *cryoconiti*, of cryoconite).

Cells are Gram-stain-negative, aerobic, motile by means of a monopolar flagellum, 1.1–3.2 μm in length and 1.0–1.1 μm in width. Colonies are round and pale yellow after 1–2 days of incubation at 28°C on Nutrient Agar plate. Growth occurs at −2°C to +32°C (optimum, 25–30°C), pH 5.0–11.0 (optimum, pH 7.0), and 0%–4.0% (w/v) NaCl (optimum, 1%). Oxidase- and catalase-positive. Hydrolyses gelatin, starch, and tributyrin, but not casein, esculin, and Tween 80. Positive for nitrate reduction beyond nitrite, citrate utilization, Voges–Proskauer test. Negative for H_2_S production, indole production, fermentation of glucose. Produces acid from L-rhamnose, sucrose, and melibiose. Assimilates glycerol, L-arabinose, D-ribose, D-galactose, D-glucose, D-fructose, D-mannose, inositol, D-mannitol, N-acetylglucosamine, sucrose, trehalose, D-arabitol, gluconate, and 2-ketogluconate. The major cellular fatty acids are C_16:0_, summed feature 3 (C_16:1_  *ω*7*c* and/or C_16:1_  *ω*6*c*), summed feature 8 (C_18:1_  *ω*6*c*/*ω*7*c*), and C_17:0_ cyclo. The genomic DNA G + C content is 58.6 mol%.

The type strain RT4P38^T^ (= CGMCC 1.24349^T^ = JCM 37137^T^) was isolated from a cryoconite sample collected from the Renlongba Glacier located in Tibet Autonomous Region, P.R. China. The GenBank accession number for the 16S rRNA gene sequence reported in this paper is PX415455. The genome sequence has been deposited at DDBJ/ENA/GenBank under the accession number JBUBUW000000000.

### Description of *Pseudomonas pasteuri* sp. nov

*Pseudomonas pasteuri* (pas.teu’ri. N.L. gen. n. *pasteuri*, of Louis Pasteur).

Cells are Gram-stain-negative, aerobic, motile by means of a monopolar flagellum, 1.3–2.7 μm in length and 0.9–1.2 μm in width. Colonies are round and pale yellow after 1–2 days of incubation at 28°C on Nutrient Agar plate. Growth occurs at −2°C to +32°C (optimum, 25°C–30°C), pH 5.0–11.0 (optimum, pH 7.0), and 0%–4.0% (w/v) NaCl (optimum, 1%). Oxidase- and catalase-positive. Hydrolyses gelatin, starch, tributyrin, and casein, but not esculin and Tween 80. Positive for reduction of nitrates to nitrites, citrate utilization, Voges–Proskauer test. Negative for H_2_S production, indole production, fermentation of glucose. Produces acid from melibiose and L-arabinose. Assimilates glycerol, L-arabinose, D-ribose, D-galactose, D-glucose, D-fructose, D-mannose, D-mannitol, N-acetylglucosamine, trehalose, D-arabitol, gluconate, and 2-ketogluconate. The major cellular fatty acids are C_16:0_, C_17:0_ cyclo, summed feature 3 (C_16:1_  *ω*7*c* and/or C_16:1_  *ω*6*c*), and summed feature 8 (C_18:1_  *ω*6*c*/*ω*7*c*). The genomic DNA G + C content is 59.0 mol%.

The type strain RT6P73^T^ (= CGMCC 1.24426^T^ = JCM 37138^T^) was isolated from a cryoconite sample collected from the Renlongba Glacier located in Tibet Autonomous Region, P.R. China. The GenBank accession number for the 16S rRNA gene sequence is PX415456. The genome sequence has been deposited at DDBJ/ENA/GenBank under the accession number JBUBUV000000000.

### Description of *Pseudomonas ketogluconatis* sp. nov

*Pseudomonas ketogluconatis* (ke.to.glu.co.na’tis. N.L. gen. n. *ketogluconatis*, of ketogluconate).

Cells are Gram-stain-negative, aerobic, motile by means of a monopolar flagellum, 1.5–2.2 μm in length and 0.8–1.0 μm in width. Colonies are round and pale yellow after 1–2 days of incubation at 28°C on Nutrient Agar plate. Growth occurs at −2°C to +30°C (optimum, 25°C–30°C), pH 5.0–11.0 (optimum, pH 7.0), and 0%–4.0% (w/v) NaCl (optimum, 1%). Oxidase- and catalase-positive. Hydrolyses tributyrin, but not starch, casein, gelatin, esculin, and Tween 80. Positive for reduction of nitrates to nitrites, citrate utilization, Voges–Proskauer test. Negative for H_2_S production, indole production, fermentation of glucose. Produces acid from D-glucose, D-mannitol, L-rhamnose, melibiose, and L-arabinose. Assimilates glycerol, L-arabinose, D-ribose, D-galactose, D-glucose, D-fructose, D-mannose, D-mannitol, D-arabitol, gluconate, 2-ketogluconate, and 5-ketogluconate. The major cellular fatty acids are C_16:0_, C_17:0_ cyclo, and summed feature 3 (C_16:1_  *ω*7*c* and/or C_16:1_  *ω*6*c*). The genomic DNA G + C content is 59.3 mol%.

The type strain GB2N2^T^ (= CGMCC 1.24744^T^ = JCM 37118^T^) was isolated from an ice sample collected from the Gawalong Glacier located in Tibet Autonomous Region, P.R. China. The GenBank accession number for the 16S rRNA gene sequence is PX415457. The genome sequence has been deposited at DDBJ/ENA/GenBank under the accession number JBUBUU000000000.

### Description of *Pseudomonas mannitolis s*p. nov

*Pseudomonas mannitolis* (man.ni.to’lis. N.L. gen. n. *mannitolis*, of mannitol).

Cells are Gram-stain-negative, aerobic, motile by means of a monopolar flagellum, 1.2–3.1 μm in length and 0.8–1.1 μm in width. Colonies are round and pale yellow after 1–2 days of incubation at 28°C on Nutrient Agar plate. Growth occurs at −2°C to +32°C (optimum, 25°C–30°C), pH 5.0–11.0 (optimum, pH 7.0), and 0%–4.0% (w/v) NaCl (optimum, 1%). Oxidase- and catalase-positive. Hydrolyses starch, tributyrin, gelatin, but not casein, esculin, and Tween 80. Positive for reduction of nitrates to nitrites, citrate utilization, Voges–Proskauer test, fermentation of glucose. Negative for H_2_S production, indole production. Produces acid from D-glucose, D-mannitol, L-rhamnose, melibiose, amygdalin, and L-arabinose. Assimilates glycerol, L-arabinose, D-galactose, D-glucose, D-mannitol, and gluconate. The major cellular fatty acids are C_16:0_, summed feature 3 (C_16:1_  *ω*7*c* and/or C_16:1_  *ω*6*c*), summed feature 8 (C_18:1_  *ω*6*c*/*ω*7*c*), and C_17:0_ cyclo. The genomic DNA G + C content is 58.7 mol%.

The type strain PB3P13^T^ (= CGMCC 1.25193^T^ = JCM 37136^T^) was isolated from an ice sample collected from the Puruogangri Glacier located in Tibet Autonomous Region, P.R. China. The GenBank accession number for the 16S rRNA gene sequence is PX415458. The genome sequence has been deposited at DDBJ/ENA/GenBank under the accession number JBUBUT000000000.

## Supplementary Material

Supplementary_material_ycag204

## Data Availability

The 16S rRNA gene sequences were deposited in GenBank under accession numbers JX949205, JX949981, JX949552, JX949553, JX949558, JX949570, JX949408, PX415443–PX415458. The GenBank/EMBL/DDBJ accession numbers for the whole-genome sequences are JBUBUT000000000-JBUBVP000000000. Proteomic data have been deposited in the ProteomeXchange Consortium via PRIDE under accession PXD073896.
