# Supplementary material for: Twenty-two novel glacier Pseudomonas species expand cultivated representation: nitrogen-cycling potential and temperature-responsive proteomes
Source: ISME Commun. 2026 Jul 12;6(1):ycag204. doi: 10.1093/ismeco/ycag204 (PMC13600387; doi:10.1093/ismeco/ycag204)
Supplement: Supplementary file 1 [file supplementary_material_ycag204.zip › Supp_ycag204.pdf]

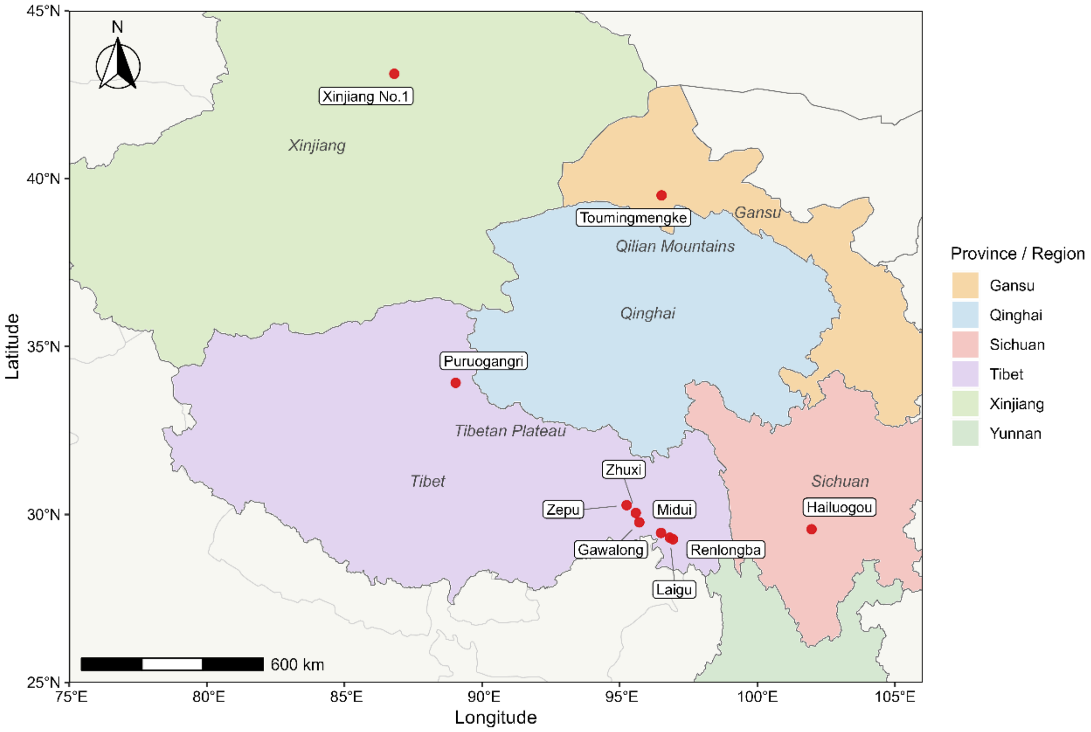

Fig. S1. Sampling locations of glacier-derived *Pseudomonas* isolates in western China.

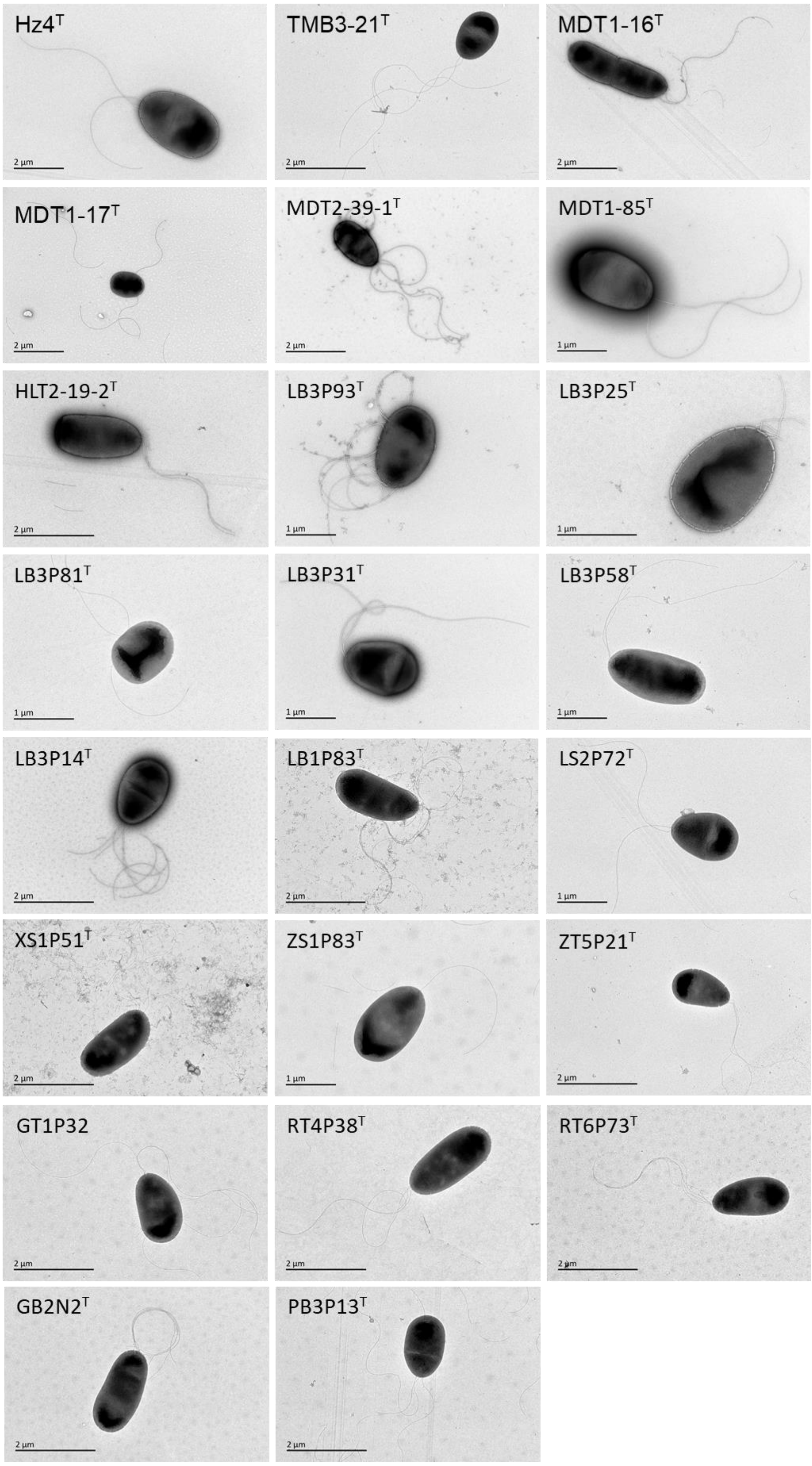

Fig. S2. Transmission electron micrographs of representative glacier *Pseudomonas* isolates. Cells are rod-shaped and flagellated, consistent with motility.

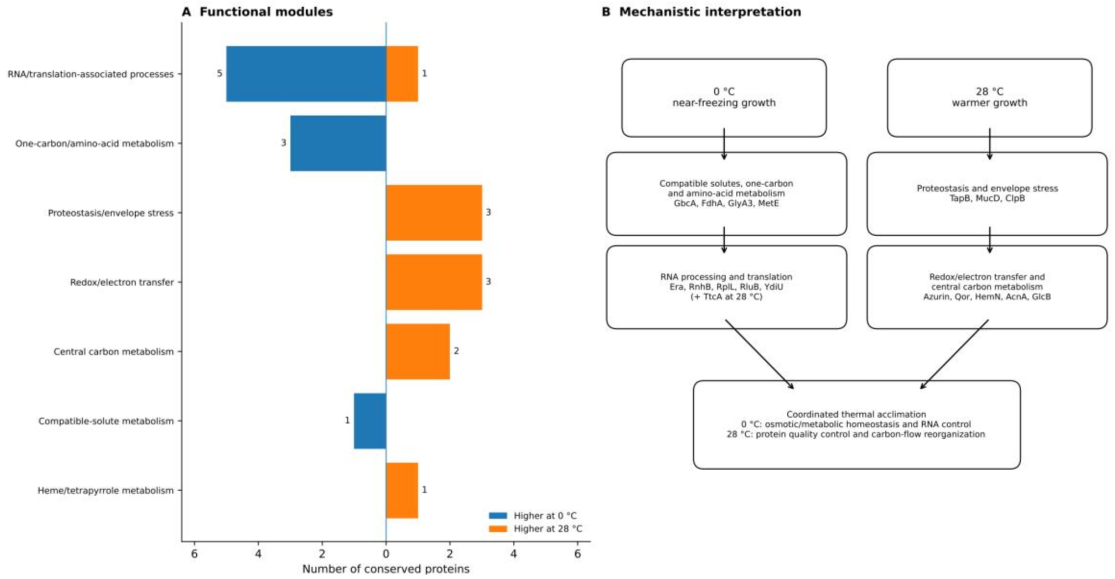

Fig. S3. Module-level summary of conserved temperature-responsive proteins across four representative glacier-derived *Pseudomonas* species.

(a) Functional-module classification of the 19 conserved temperature-responsive proteins identified in at least three of the four representative species. Bars indicate the number of proteins with higher abundance at 0 °C or 28 °C. (b) Schematic interpretation of the conserved temperature-responsive modules. Proteins with higher abundance at 0 °C were mainly associated with compatible-solute metabolism, one-carbon/amino-acid metabolism, and RNA/translation-associated processes, whereas proteins with higher abundance at 28 °C were mainly associated with proteostasis, redox/electron-transfer processes, heme/tetrapyrrole metabolism, and central carbon metabolism. These patterns suggest coordinated adjustment of osmotic/metabolic homeostasis, RNA/translation control, protein quality control and carbon-flow reorganization during thermal acclimation.

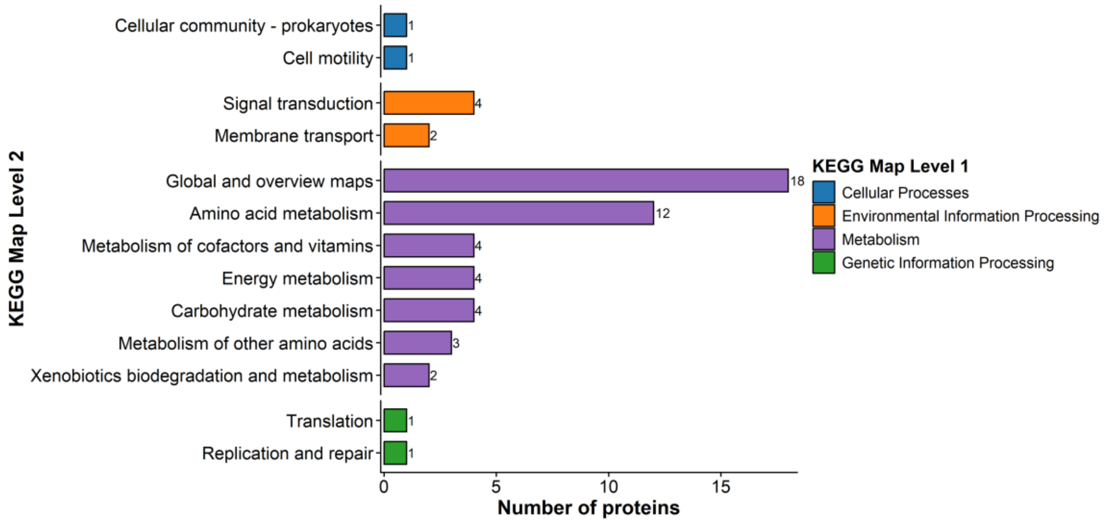

Fig. S4. KEGG functional composition of temperature-responsive proteins identified by the temperature main-effect model.

Bar plot showing the number of proteins assigned to each KEGG level-2 category among proteins exhibiting a significant temperature main effect ($P < 0.05$) and an absolute abundance change of ≥3-fold ($|log2FC| \geq log2(3)$) between 0 °C and 28 °C. Bar colours indicate the corresponding KEGG level-1 functional class (Cellular Processes, Environmental Information Processing, Metabolism, and Genetic Information Processing). Numbers at the bar ends denote protein counts per category.
